# Candidate regulators of drought stress in tomato revealed by comparative transcriptomic and proteomic analyses

**DOI:** 10.3389/fpls.2023.1282718

**Published:** 2023-10-23

**Authors:** Minmin Liu, Gangjun Zhao, Xin Huang, Ting Pan, Wenjie Chen, Mei Qu, Bo Ouyang, Min Yu, Sergey Shabala

**Affiliations:** ^1^ International Research Centre for Environmental Membrane Biology and Department of Horticulture, Foshan University, Foshan, China; ^2^ Guangdong Key Laboratory for New Technology Research of Vegetables, Vegetable Research Institute, Guangdong Academy of Agricultural Sciences, Guangzhou, China; ^3^ Key Laboratory of Horticultural Plant Biology (Ministry of Education), Huazhong Agricultural University, Wuhan, China; ^4^ School of Biological Science, University of Western Australia, Crawley, WA, Australia

**Keywords:** RNA-seq, proteomics, drought stress, *ABA-response element binding factor*, AREB1, heat shock protein, HSP

## Abstract

Drought is among the most common abiotic constraints of crop growth, development, and productivity. Integrating different omics approaches offers a possibility for deciphering the metabolic pathways and fundamental mechanisms involved in abiotic stress tolerance. Here, we explored the transcriptional and post-transcriptional changes in drought-stressed tomato plants using transcriptomic and proteomic profiles to determine the molecular dynamics of tomato drought stress responses. We identified 22467 genes and 5507 proteins, among which the expression of 3765 genes and 294 proteins was significantly changed under drought stress. Furthermore, the differentially expressed genes (DEGs) and differentially abundant proteins (DAPs) showed a good correlation (0.743). The results indicated that integrating different omics approaches is promising in exploring the multilayered regulatory mechanisms of plant drought resistance. Gene ontology (GO) and pathway analysis identified several GO terms and pathways related to stress resistance, including response to stress, abiotic stimulus, and oxidative stress. The plant hormone abscisic acid (ABA) plays pivotal roles in response to drought stress, *ABA-response element binding factor* (*AREB*) is a key positive regulator of ABA signaling. Moreover, our analysis indicated that drought stress increased the abscisic acid (ABA) content, which activated *AREB1* expression to regulate the expression of *TAS14*, *GSH-Px-1*, and *Hsp*, ultimately improving tomato drought resistance. In addition, the yeast one-hybrid assay demonstrated that the AREB1 could bind the Hsp promoter to activate Hsp expression. Thus, this study involved a full-scale analysis of gene and protein expression in drought-stressed tomato, deepening the understanding of the regulatory mechanisms of the essential drought-tolerance genes in tomato.

## Introduction

Drought is arguably the most severe environmental constraint limiting crop growth, development, yield and product quality (Gupta et al., 2020; Liu et al., 2020). Therefore, understanding how plants recognize and transmit drought signals to cellular machinery to activate tolerance responses is critical for sustainable agriculture (Mishra and Singh, 2010). Drought induces osmotic, oxidative, and nutritional stresses in plants, and as a result, plants evolved complex drought tolerance mechanisms involving a plethora of physiological, biochemical, and morphological adaptations.

Plants respond to drought stress by regulating leaf wax thickness, pubescence, and stomatal development and activities and altering their root structure, hormonal balance, and redox status (Ilyas et al., 2021; Liu et al., 2017). Plants also activate *de novo* synthesis of compatible solutes to osmotically adjust to low water conditions in the soil. These adaptive processes involve the expression of numerous genes via a complex regulatory network. This regulation involves the perception and transmission of the stress signal and activation of stress response genes, which change the physiological and biochemical activities of plants to adapt to stress (Zhu, 2016). Abscisic acid (ABA) is classified as a stress hormone and plays an important role in plant responses to abiotic stress (Yoshida et al., 2014). Specifically, ABA-dependent protein AREB1 plays an important role in regulating drought responses by activating the expression of downstream stress-related target genes (Zhu, 2016).

Tomato (*Solanum lycopersicum*) is an important vegetable crop widely cultivated worldwide; however, its commercial production is limited by abiotic stresses such as drought. Drought stress reduces tomato yield and fruit quality (Pervez et al., 2009; Liang et al., 2020). Tomato fruit is rich in nutrients, including lycopene, iron, vitamin A, vitamin C, and antioxidants (Sato et al., 2012). Therefore, identifying the drought-resistance genes in tomato and determining their functional mechanisms is important for the sustainability and profitability of tomato production systems.

The “omics” approach is a powerful and effective tool for exploring stress-responsive genes (Zhuang et al., 2014). For example, RNA sequencing (RNA-seq), has been successfully applied to identify stress-responsive genes in different plant species, including *Arabidopsis*, rice, tomato, pepper, and corn (Bechtold et al., 2016; Liu et al., 2017; Liu et al., 2017; Wilkins et al., 2016; Liu et al., 2018). Besides, several studies have used proteomics to understand the stress resistance mechanisms of plants because plant stress responses directly involve proteins (Wang et al., 2016; Kosová et al., 2018).

Proteomics has uncovered many proteins involved in drought responses, including ribose-1,5-diphosphate carboxylase (Rubisco), chlorophyll-binding protein a/b, sucrose synthase, and UDP glucose-6-dehydrogenase (related to carbon metabolism). These proteins participate in drought response pathways in *Arabidopsis*, rice, and corn (Liu and Bennett, 2011; Ahmad et al., 2016). Tandem mass tag (TMT)-based proteomics is a typical shotgun analysis that couples TMT with liquid chromatography-tandem mass spectrometry (LC-MS/MS) to reveal the function, expression, and other aspects of plant proteins. The advantages of this approach outweigh traditional protein determination methods due to its high reproducibility, multiplexing capacity, and high accuracy (Zhang and Elias, 2017; Navarrete-Perea et al., 2018).

Given the complexity of drought stress effects, there is no “silver bullet” that can be used to alleviate drought stress, and different species may rely on different sets of sub-traits to adapt to drought (Farooq et al., 2009; Ilyas et al., 2021). The recent emergence of novel protein analysis methods has contributed to the understanding of tomato drought tolerance mechanisms (Bolger et al., 2014a; Liang et al., 2020). Tamburino et al. (2017) used 2D-DIGE-MS/MS to investigate the expression of tomato proteins in the chloroplast. The study found that tomato responds to drought stress through a specific chloroplast-to-nucleus signaling interconnection with the ABA signaling network. Moreover, Çelik et al. (2021) used the 2-DE proteomic approach to demonstrate that the proteins involved in carbohydrate and energy metabolism, stress responses and defense, transcription, and phosphorylation enhance drought tolerance. Zhou et al. (2013) revealed root water deficit stress-related proteins, such as hydrophilic proteins and calmodulin, that conferred drought resistance in tomato. Furthermore, Rai et al. (2021) used the two-dimensional gel electrophoresis to identify and quantify the proteomic responses of tomato leaves to drought stress. The study found that most of the proteins whose levels had changed after drought stress substantially correlated with defense responses, oxidative stress, detoxification, protein synthesis, and energy and carbon metabolism. However, to the best of our knowledge, the integrative transcriptomic and proteomic analysis to investigate the drought tolerance genes in tomato leaves under drought stress has been not conducted.

Drought tolerance genes function differently in different plants. For example, while there is a certain correlation between drought stress and aquaporin gene expression in plants, the response of aquaporin to drought varies greatly among different plant tissues, species, varieties, or at different stress levels. Aquaporins operate as water channels to control water transport in and out of the cells and play an important role in improving the growth and adaptation of crops under drought stress (Ding et al., 2016). Overexpressing tomato aquaporin (tonoplast intrinsic protein2, TIP2) and that of *Thellungiella salsuginea* (tonoplast intrinsic protein1, TIP1) increased water deficit tolerance of *A. thaliana*, while those of *Glycine soja* (TIP2) and *Triticum aestivum* (TIP1) did not (Peng et al., 2007; Sade et al., 2009; Wang et al., 2011; Wang et al., 2013; Xu et al., 2013). Another example of proteins involved in stress plant stress responses is annexins. Annexins act as sensors of calcium signals in plant cells and play important roles in plant stress tolerance and development (Huh et al., 2010). Annexins have been shown to be strongly up-regulated in *Arabidopsis* under drought stress, and their overexpression improved the drought tolerance of *Arabidopsis* (Konopka-Postupolska et al., 2009). However, expression of annexins in rice was downregulated under a 3-h dehydration stress (Singh et al., 2014). The 70 kDa heat shock proteins (HSP70s) act as primary chaperones in the folding process of newly synthesized proteins and play a crucial role in drought stress responses in plants (ul Haq et al., 2019). Maize Hsp70 was up-regulated during drought stress (Benešová et al., 2012), while chickpea Hsp70 proteins were first down-regulated in the early stage of growth in drought-tolerant cultivars (ul Haq et al., 2019). These results show the complexities of different signaling pathways involved in drought stress responses in different plants. Thus, it is speculated that tomato drought tolerance mechanisms are not entirely the same as those of other plants. This study characterized the transcriptomic and proteomic responses of tomato under drought stress by analyzing the effects of drought stress on the physiological, transcriptional, and post-transcriptional responses of tomato under drought and natural soil conditions. For doing this, we measured the chlorophyll fluorescence parameters and malondialdehyde (MDA), hydrogen peroxide (H_2_O_2_), proline, soluble sugar, and ABA contents of control and of drought stress tomato leaves. RNA-Seq and tandem mass tags-based (TMT) proteomics were used to analyze differentially expressed genes and proteins, and the functional analysis of RNA-Seq and TMT data was done using bioinformatics methods. Finally, the yeast one-hybrid assay was conducted to demonstrate the interaction between AREB1 and Hsps. Our results showed that drought stress affected tomato growth. There was a poor correlation between transcripts and the abundance of their corresponding proteins under drought stress conditions; however, the significantly differentially expressed genes and proteins had a good correlation. Gene Ontology and pathway analysis revealed several drought stress-related genes. The ABA content increased, which in turn activated AREB1 to regulate the expression of genes such as Hsps, TAS, and ARS, thereby improving plant drought tolerance under drought stress. The correlation of transcript-to-protein abundance may provide necessary information to understand the drought tolerance mechanism of tomato.

## Materials and methods

### Plant materials and drought treatment

Tomato (*Solanum lycopersicum*; cultivar M82) seeds were obtained from the Tomato Genetic Resource Center, University of California-Davis, CA, USA. Uniformly-sized seeds were germinated in a petri dish containing moist filter paper in the dark at 28°C. Evenly germinated seeds were then sown in a plastic bowl (7 × 7 × 7 cm) containing vermiculite, peat, and perlite (1:1:1). The seedlings were kept in a growth chamber at 25 ± 2°C, with a light/dark cycle of 16 h/8 h. Seedling management was conducted according to the method by Liu et al. (2017).

At the five-leaf stage, the drought-stress treatment was performed by withholding water for the treatment plants, and the control plants were irrigated daily. After 7 days of drought stress, when the soil water content reached ca 18%, the third leaf from the bottom of each seedling was collected, quickly frozen in liquid nitrogen, and stored at -70°C until use. The experiment was conducted in triplicate, with 10 seedlings per replicate.

### Relative water content

The relative water content (RWC) of leaves was measured, as described by Tuna et al. (2007). Briefly, fresh leaves were sampled from plants, and their fresh weight (FW) was measured. Thereafter, the leaves were immersed in distilled water for 24 h in the dark and weighed to measure the turgid weight (TW). The leaves were then oven-dried at 80°C until a constant weight was achieved to calculate the dry weight (DW). RWC was calculated as follows:


RWC = (FW−DW)/(TW−DW)×100

### H_2_O_2_ content

Powdered leaf samples (2 g) were homogenized with 5 mL of chilled acetone and centrifuged at 1000 g for 20 min at 4°C. The supernatant was immediately used to analyze the H_2_O_2_ content using the detection kits (Cat. No. A064-1-1, Nanjing Jiancheng Bioengineering Institute), according to the manufacturer’s instructions (Huang et al., 2013).

### Soluble sugar content

The soluble sugar content of tomato leaves was measured as described by Mustroph et al. (2006). Briefly, 0.5 g of fresh samples were extracted in 80% ethanol by stirring continuously at 80°C for 2 h. After the ethanol evaporated, distilled water was added to re-dissolve the residue. Thereafter, 0.15% anthrone solution was added to each sample, and the mixture was heated at 95°C for 15 min, followed by cooling at room temperature. The absorbance of the reaction solution was measured at 620nm.

### MDA content

The MDA content was measured according to the method by Hodges et al. (1999). Briefly, 0.3 g of leaf tissues was homogenized in 5 mL of 5% (w/v) trichloroacetic acid and centrifuged at 12,000 g for 15 min. The supernatant was mixed with an equal volume of thiobarbituric acid (0.5% in 20% [w/v] trichloroacetic acid) and heated at 95°C for 25 min. The mixture was then centrifuged at 7500 g for 5 min, and the absorbance of the supernatant was measured at 532 nm and corrected by subtracting the A600 value.

### Proline content

The proline content of tomato leaves was determined using the method by Bates et al. (1973). Briefly, fresh leaf tissues (0.5 g) were extracted in 5 mL of 3% sulfosalicylic acid at 95°C for 15 min. After filtration, 2 mL of the supernatant was transferred to a new tube containing 2 mL of acetic acid and 2 mL of acidified ninhydrin reagent. After incubation at 95°C for 30 min, 5 mL of toluene was added to the samples under sufficient oscillation to extract the red product. The absorbance of the toluene layer was measured at 532nm.

### ABA content

ABA content of tomato leaves was determined using the method by Liu et al. (2012). Briefly, 20 mg of freeze-dried leaf samples were extracted twice using 750 μL of plant hormone extraction buffer (80:19:1 of methanol: water: glacial acetic acid; v/v/v) containing 20 ng/mL ^2^H_6_ABA as the internal standard. Quantitative analysis was conducted in ABI 4000 Q-Trap equipment (Applied Biosystems).

### Measurements of chlorophyll fluorescence parameters

Photosynthesis is very sensitive to drought stress, and chlorophyll fluorescence parameters are widely used to determine stress responses in plants. Tomato plants were kept in the dark for 30 min before measuring various chlorophyll fluorescence characteristics such as quantum yield of photosystem II (Y(II)), maximum quantum efficiency of photosystem II (Fv/Fm), and electron transport rate (ETR)] of the leaves of the control and drought-stressed plants using a modulated chlorophyll fluorescence imaging system (IMAGING-PAM, WALZ, Germany). All the measurements were performed on the fourth lower leaf of each seedling.

### RNA isolation and library preparation

Total RNA was isolated using TRIzol reagent (Invitrogen, CA, USA) according to the manufacturer’s instructions, and the RNA quality was checked via 1% agarose gel electrophoresis, as described by Liu et al. (2017). RNA integrity and concentration were further analyzed using the Agilent 2100 Bioanalyzer (Agilent Technologies, CA, USA). The RNA was reverse transcribed to cDNA, which was then sequenced on the HiSeq 2500 platform using the 150 bp paired-end method.

### Identification of differentially expressed genes

The differentially expressed genes (DEGs) were identified, as described in our previous study (Liu et al., 2017). Briefly, raw reads were processed using Trimmomatic (Bolger et al., 2014b) and the clean reads were assembled based on the tomato Heinz 1706 reference genome (version 3.0) using Hisat2 (Kim et al., 2019). DESeq2 (Love et al., 2014) was used to identify the DEGs based on the |log2(fold change) | ≥ 1 and padj < 0.05 threshold.

### Protein extraction

Leaf samples (500 mg) were ground in liquid nitrogen and mixed with lysis buffer (1% TritonX-100, 10 mM dithiothreitol, and 1% protease inhibitor cocktail) in 5 mL centrifuge tubes. The mixture was sonicated for 3 min on ice using a high-intensity ultrasonic processor (Scientz, China), and an equal volume of phenol (pH 8.0) was added. The mixture was vortexed for 5 min and centrifuged at 5000 g for 10 min at 4°C. The upper phenol phase was transferred to new centrifuge tubes, and four volumes of ammonium sulfate-saturated methanol were added, followed by incubating the mixture at -20°C for at least 6 h to precipitate the protein. After centrifuging the mixture at 5000 g for 10 min at 4°C, the supernatant was discarded, and the remaining precipitate was washed thrice with ice-cold methanol, followed by ice-cold acetone. The protein precipitate was re-dissolved in 8 M urea, and the protein concentration was determined using the Bradford method.

### Digestion and TMT labeling

The protein solution was diluted to 5 mM and digested at 56°C for 30 min, after which iodoacetamide was added to the digested protein to a final concentration of 11 mM. The mixture was then incubated at room temperature for 15 min in the dark. Finally, the sample was diluted to < 2 M urea before adding trypsin at a 1:50 (trypsin: protein) mass ratio for an overnight enzymatic hydrolysis at 37°C. Trypsin was added again to the mixture at a 1:100 (trypsin: protein) mass ratio and left for 4 h for further enzymatic hydrolysis. The hydrolysis was to ensure the complete breakdown of proteins into smaller fragments for easy analysis and identification of the proteins.

After trypsin digestion, the hydrolyzed peptides were desalted with strata x C18 (Phenomenex, USA), vacuum lyophilized, dissolved in 0.5 M TEAB, and labeled using the TMT kit (Thermo Fisher Scientific, USA), following the manufacturer’s instructions. The TMT kit enables multiplex relative quantitation by mass spectrometry (MS), with good reproducibility, high sensitivity, wide protein coverage, and the ability to identify many proteins.

### HPLC fractionation and LC-MS/MS analysis

The tryptic peptides were separated into fractions by a high pH reverse-phase HPLC (high-performance liquid chromatography) using Agilent 300 Extend C18 column (5 μm particles, 4.6 mm ID, 250 mm length). The peptides were separated with a gradient of 8% to 32% acetonitrile in 10 mM ammonium bicarbonate (pH 9) over 54 min. Thereafter, the peptides were combined into 9 fractions and dried by vacuum centrifugation.

The samples were dissolved in solvent A (0.1% formic acid, 2% acetonitrile/water) and directly loaded onto a homemade reversed-phase analytical column (25 cm length, 100 μm). The peptides were then separated using a 30 min gradient from 8 to 16% solvent B (0.1% formic acid in 90% acetonitrile), followed by 25 min from 16 to 30% and 80% for 2 min, and held at 80% for 3 min. The flow rate was 400 nL/min in an EASY-nLC 1000 UPLC system (Thermo Fisher Scientific, USA).

The separated peptides were analyzed in Orbitrap FusionTM LumosTM (Thermo Fisher Scientific, USA) with a nano-electrospray ion source at 2.0 kV. The full MS scan resolution was set to 60,000 for a 350–1550 m/z scan range. Up to 20 most abundant precursors were then selected via a 30 s dynamic exclusion for further MS/MS analyses. The HCD (Higher Energy Collision Dissociation) fragmentation was performed at a 32% normalized collision energy (NCE), and the fragments were detected in the Orbitrap at 15,000 resolutions, with the fixed first mass set at 100 m/z. The automatic gain control (AGC) target was set at 5E4, with a 5E4 intensity threshold and 60 ms maximum injection time.

### Database search

The resulting MS/MS data were processed using the Maxquant search engine (v.1.5.2.8). The tandem mass spectra were searched against the tomato Heinz 1706 reference genome version 3.0 (http://solgenomics.net) concatenated with the reverse decoy database. The mass tolerance for precursor ions was 20 ppm and 5 ppm in the First search and Main search, respectively, with 0.02 Da mass tolerance for fragment ions. Cysteine (Cys) carbamidomethylation was specified as fixed modification, while methionine (Met) oxidation was set as variable modification. The false discovery rate (FDR) was < 1%, and the minimum peptide score was > 40.

### Bioinformatics analysis

Gene Ontology (GO) and pathway analysis were conducted to clarify the functional and gene pathway enrichment of DEGs. The GO annotation of the proteome was derived from the UniProt-GOA database (http://www.ebi.ac.uk/GOA/). Pathway analysis was conducted using the KEGG database (http://www.genome.jp/kegg) (Kanehisa et al., 2013). Furthermore, Fisher’s exact test was used to determine whether the DEGs or differentially expressed proteins (DEPs) were significantly enriched in the GO terms/KEGG pathways. A P-value of < 0.05 in the enrichment test was considered significant. Moreover, we used Wolfpsort, a subcellular localization tool, to predict the subcellular localization of the proteins (Horton et al., 2007). The DEPs with a P-value of < 0.05 and a fold change of > 1.3 were considered significantly up-regulated, while those with a P-value of < 0.05 and a fold change of < 1/1.3 were considered significantly down-regulated(Li et al., 2018). The DEPs were searched against the STRING database (version 10.1) for protein-protein interactions (confidence score>0.7) (Szklarczyk et al., 2014), and the COG/KOG protein functional annotation was conducted using KOG sequence alignment in the NCBI database.

## Results

### Effects of drought stress on tomato

**Figure 1:**
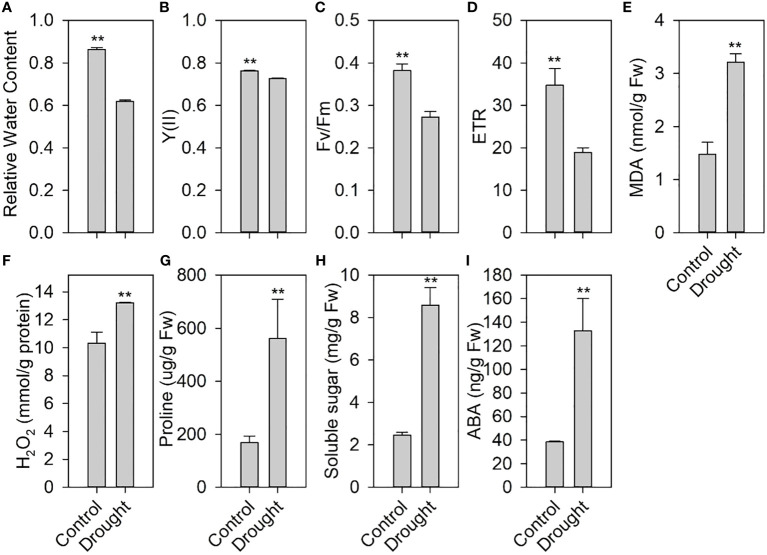
Effects of drought stress on physiological and biochemical indexes of tomato. **(A)** Relative water content in leaves after drought stress. **(B)** Quantum yield of photosystem II, Y(II). **(C)** Maximum quantum efficiency of photosystem II, Fv/Fm. **(D)** Electron transfer rate, ETR. **(E)** Malondialdehyde (MDA) content. **(F)** H_2_O_2_ content. **(G)** Proline content. **(H)** Soluble sugar. **(I)** Abscisic acid (ABA) content in leaves under drought stress. ** indicates a significant difference at P < 0.01 using two-tailed Students t-test.

We measured the physiological and biochemical indicators of drought stress during the growth and development of tomato. After 7 days of drought treatment, the RWC of the treated leaves was significantly lower than that of the control (**Figure 1A**). Moreover, drought stress significantly decreased the chlorophyll fluorescence parameters, including Y(II) (**Figure 1B**), Fv/Fm (**Figure 1C**), and ETR (**Figure 1D**). Drought stress increased the contents of MDA (**Figure 1E**), an indicator of membrane lipid peroxidation, and H_2_O_2_ (**Figure 1F**), one of the main reactive oxygen species (ROS).

This further reduced the plant water status and photosynthesis and increased the membrane lipid peroxidation of tomato, thereby affecting tomato normal growth and development. However, drought stress significantly increased the contents of proline (**Figure 1G**), soluble sugars (**Figure 1H**), and ABA (**Figure 1I**), which play an important role in drought tolerance.

### Differentially expressed genes and functional annotation of drought-stressed tomato

**Figure 2:**
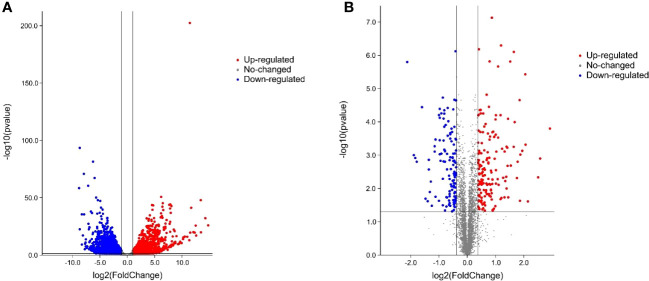
Volcano plot of differentially expressed genes **(A)** and proteins **(B)** under drought stress.

Six cDNA libraries (ContM82-1, ContM82-2, ContM82-3, DM82-1, DM82-2, and DM82-3) were constructed using total RNA extracted from the drought-treated tomato variety M82 (DM82) and the control (ContM82) and were sequenced to identify the drought-responsive genes in tomato. Each sample generated 5.87 to 8.84 Gb of raw sequence data. Low-quality raw reads and adaptor sequences were discarded, and 62.50, 54.28, 54.27, 42.61, 56.94, and 54.52 million clean reads were obtained for ContM82-1, ContM82-2, ContM82-3, DM82-1, DM82-2, and DM82-3, respectively. The clean reads were 94.99, 94.14, 93.75, 93.94, 93.58, and 94.36% mapped to the tomato reference genome (https://solgenomics.net) (**Supplementary Table S1**). Gene annotation and expression analysis detected 22,467 genes (**Figure 2A**; **Supplementary Table S2**), including 3765 DEGs (|log2 fold-change| ≥ 1 and q-value ≤ 0.05) (1527 up-regulated and 2238 down-regulated) after the drought stress (**Figure 2A**).

**Figure 3:**
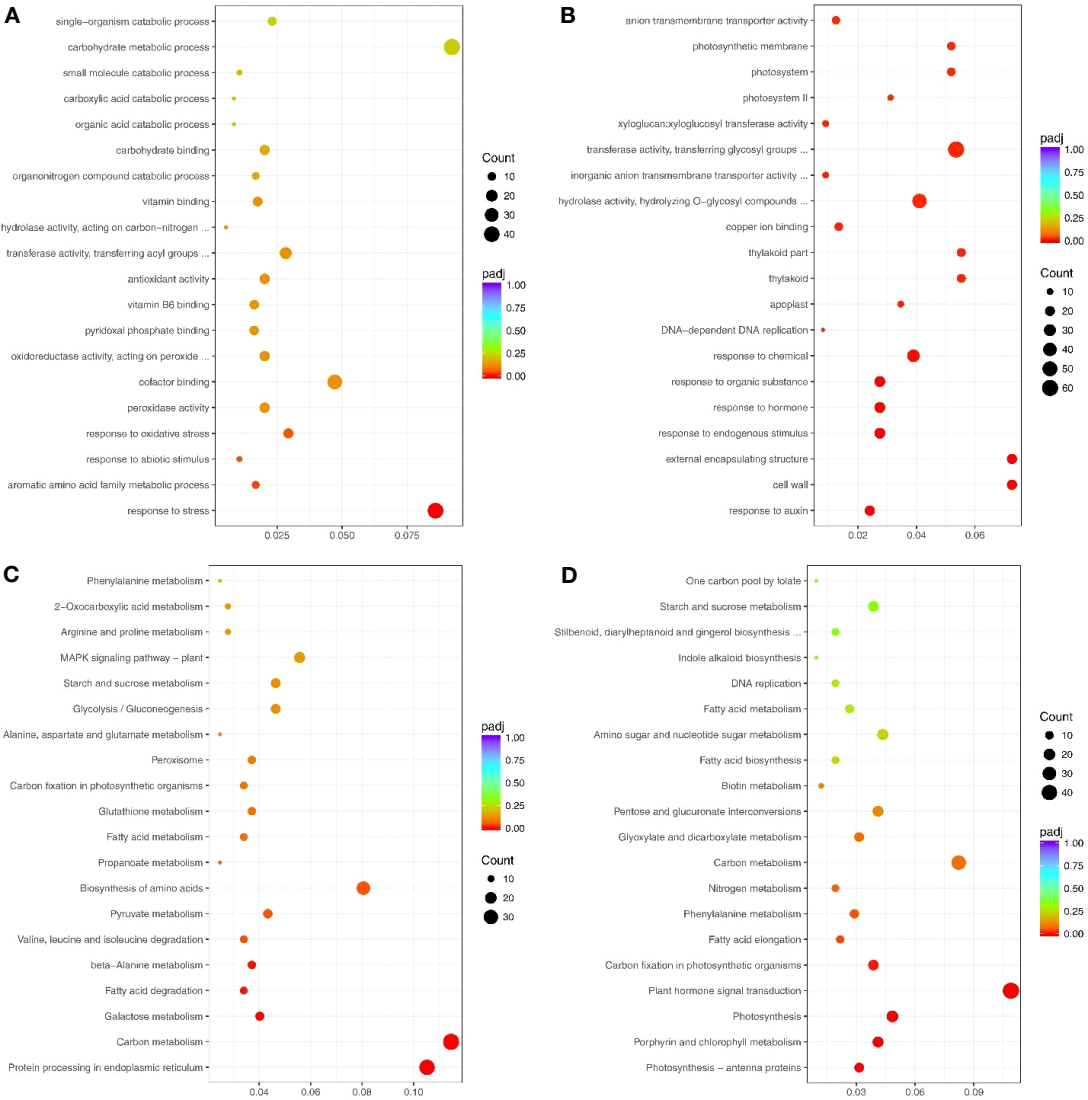
Top 20 GO terms and pathways enriched by the drought response genes. **(A)** Up- and **(B)** Down-regulated genes that enriched the GO terms. **(C)** Up- and **(D)** Down-regulated genes that enriched the pathways. Count indicates enriched differentially-expressed gene numbers; *Color scale* indicates q value.

The significantly up-and down-regulated genes (under drought treatment) were enriched in GO terms (**Supplementary Tables S3**, **S4**). The up-regulated genes under drought stress were significantly enriched in 4 GO terms: response to stress (GO: 0006950), aromatic amino acid family metabolic process (GO: 0009072), response to abiotic stimulus (GO: 0009628), and response to oxidative stress (GO:0006979) (**Figure 3A**). Conversely, the down-regulated genes under drought stress were significantly enriched in 34 GO terms, including response to auxin (GO: 0009733), response to hormone (GO: 0009725), photosystem (GO: 0009521), and enzyme inhibitor activity (GO: 0004857) (**Figure 3B**).

Most enriched KEGG pathways of the up-regulated genes under drought stress were related to abiotic stress and included MAPK signaling pathway-plant (sly04016), peroxisome (sly04146), and arginine and proline metabolism (sly00330) (**Figure 3C**; **Supplementary Table S5**). The down-regulated genes were mainly enriched in pathways related to growth and development, including photosynthesis (sly00195), plant hormone signal transduction (sly04075), and nitrogen metabolism (sly00910) (**Figure 3D**; **Supplementary Table S6**).

Overall, 3765 DEG (1527 up-regulated and 2238 down-regulated) were identified in tomato under drought stress. The GO and pathway analyses revealed that much of the significantly down-regulated genes were related to plant growth, and the significantly up-regulated genes were important for plant drought adaptation. Plants have developed several protective mechanisms during their long-term evolution, including morphological, physiological, and biochemical changes, to resist harsh external environments, such as drought conditions, at the cellular and molecular levels. These regulatory processes rely on these DEGs to form a complex regulatory network, which induces physiological and biochemical changes for surviving adversity.

### Differentially expressed proteins and drought stress response in tomato

Comparative proteomics identified 5507 proteins in ContM82 and DM82 (**Figure 2B**; **Supplementary Table S7**), which included 294 differentially abundant proteins (DAPs) |fold-change| >1.3 (mean value of all compared groups) (165 up-regulated and 129 down-regulated) (**Figure 2B**).

**Figure 4:**
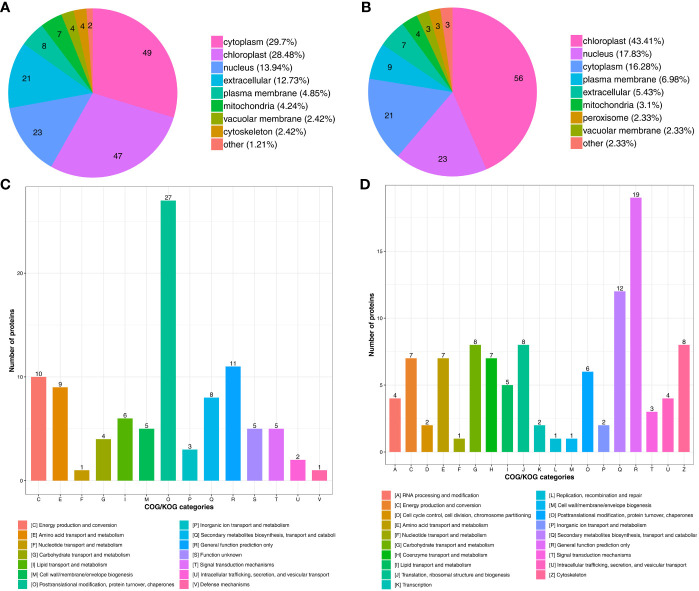
Functional classification of DAPs under drought stress. Subcellular localization chart of **(A)** Up- and **(B)** Down-regulated proteins. COG/KOG functional classification chart of **(C)** Up- and **(D)** Down-regulated proteins.

To characterize the biological functions of the DAPs under drought stress, we conducted subcellular localization and COG/KOG (Clusters of Orthologous Groups of proteins) functional classification pathway analysis. The subcellular localization prediction showed that most proteins up-regulated under drought stress were located in the cytoplasm (49 DAPs, 29.7%), chloroplast (47 DAPs, 28.48%), nucleus (23 DAPs, 13.94%), and extracellular (21 DAPs, 12.73%) (**Figure 4A**). Besides, some proteins were located in the plasma membrane (8 DAPs, 4.85%), mitochondria (7 DAPs, 4.24%), vacuolar membrane (4 DAPs, 2.42%), and cytoskeleton (4 DAPs, 2.42%) (**Figure 4A**; **Supplementary Table S8**).

Nearly half of the down-regulated proteins were located in the chloroplast (56 DAPs, 43.41%) (**Figure 4B**), followed by the nucleus (23 DAPs, 17.83%), cytoplasm (21 DAPs, 16.28%), plasma membrane (9 DAPs, 6.98%), and extracellular (7 DAPs, 5.43%). The mitochondria (4 DAPs, 3.1%), peroxisome (3 DAPs, 2.33%), and vacuolar membrane (3 DAPs, %) also contained some of the down-regulated proteins (**Figure 4B**; **Supplementary Table S9**). These results indicate that subcellular localization can predict the specific location of the DAPs within the cell, providing research directions for understanding how proteins function during drought stress.

Drought stress also up-regulated proteins related to signal transduction mechanisms, defense mechanisms, posttranslational modification, protein turnover, and chaperones (**Figure 4C**; **Supplementary Table S10**). Drought stress also down-regulated proteins involved in transcription, translation, and metabolism through processes such as the cytoskeleton, translation, ribosomal structure and biogenesis, and carbohydrate transport and metabolism (**Figure 4D**; **Supplementary Table S11**). The function and specific metabolic pathways of proteins can be determined through COG/KOG analysis, providing a reference for further research on the roles of the DAPs in tomato under drought stress.

**Figure 5:**
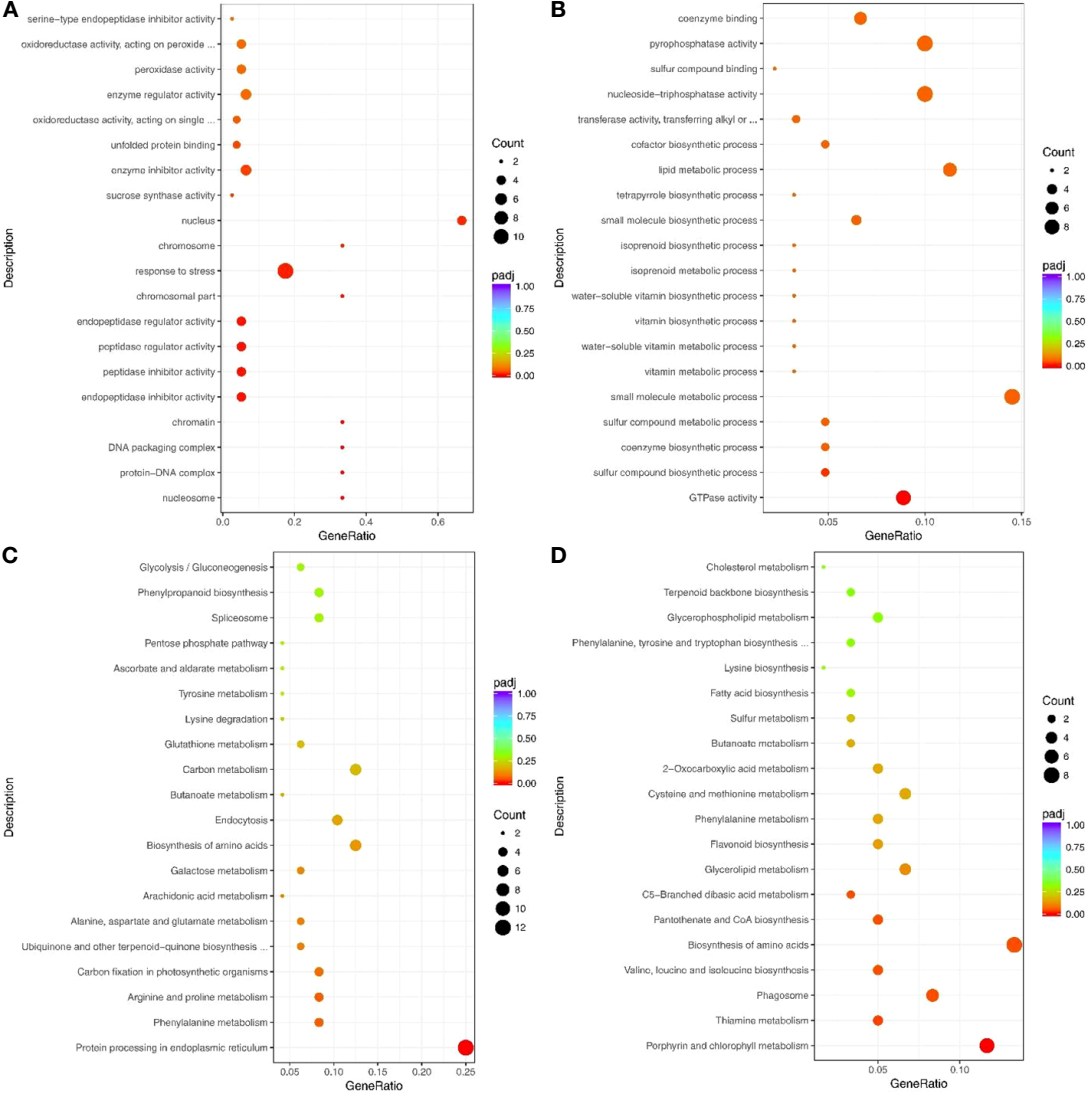
Top 20 GO terms and pathways enriched by drought response proteins. **(A)** Up- and **(B)** Down-regulated proteins that enriched the GO terms. **(C)** Up- and **(D)** Down-regulated proteins that enriched the pathways. Counts indicate the number of differentially expressed genes (DEGs); The color scale indicates the q value.

The up-regulated proteins were enriched in the GO terms related to biological process and abiotic stress, such as response to stress (GO: 0006950), response to abiotic stimulus (GO: 0009628), and oxidation-reduction process (GO: 0055114) (**Figure 5A**; **Supplementary Table S12**).

Notably, the up-regulated proteins under drought stress were enriched in many peptide-related GO terms, such as peptidase regulator activity (GO: 0061134), peptidase inhibitor activity (GO: 0030414), endopeptidase regulator activity (GO: 0061135), and endopeptidase inhibitor activity (GO: 0004866) (**Figure 5A**). The down-regulated proteins were mainly enriched in the GO terms for growth and metabolism, including GTPase activity (GO:0003924), coenzyme biosynthetic process (GO:0009108), and single-organism biosynthetic process (GO: 0044711) (**Figure 5B**; **Supplementary Table S13**). In addition, 53 up-regulated and 45 down-regulated proteins were significantly enriched in the KEGG pathways (**Figures 5C, D**, **Supplementary Table S14**, **S15**).

### Quantitative comparisons and network analysis of the DAPs and DEGs

**Figure 6:**
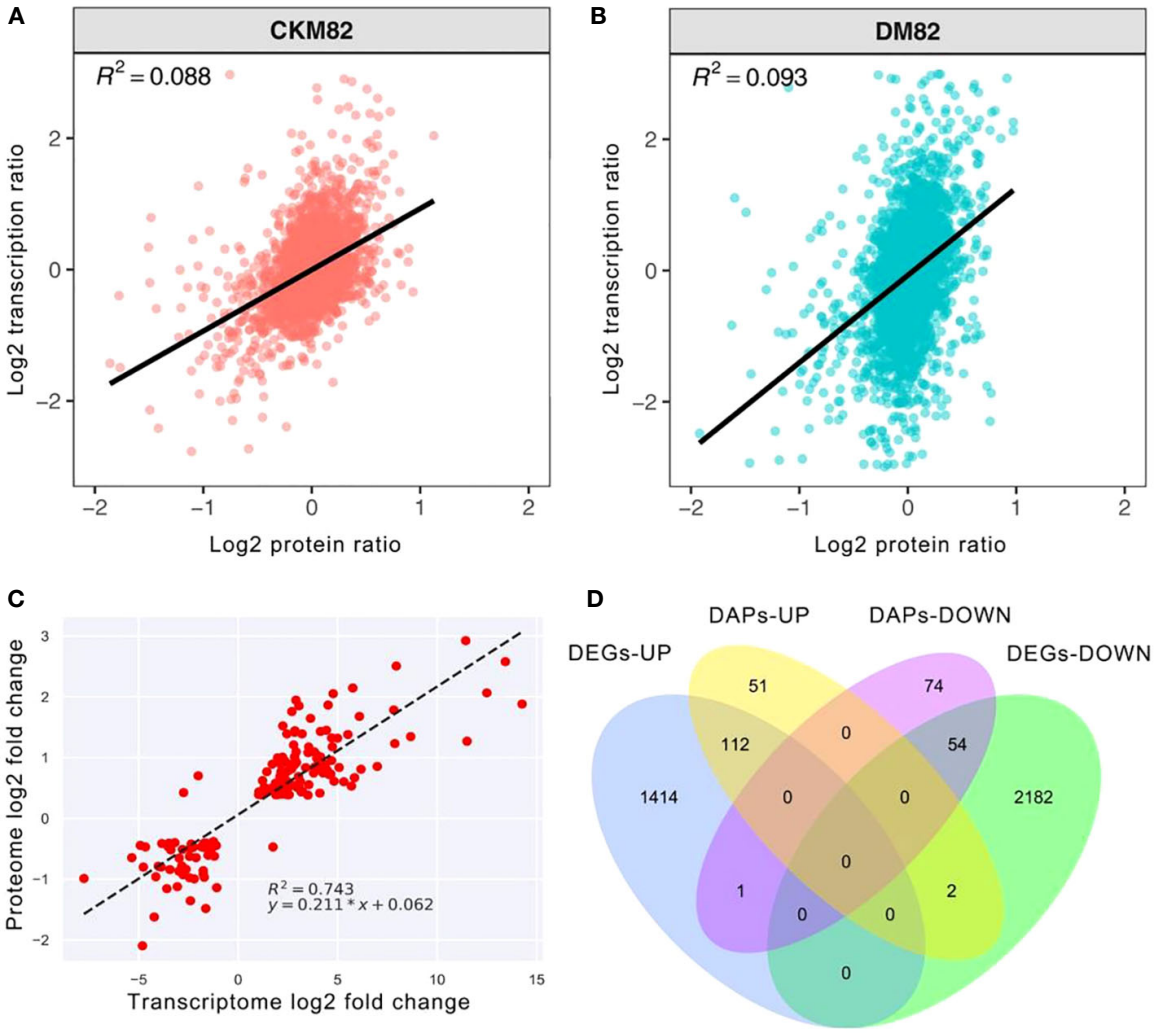
Comparing protein abundance and gene expression levels based on high throughput sequencing. Scatter plots of transcripts and their corresponding proteins **(A)** ContM82 and **(B)** DM82. **(C)** Correlations between transcript levels and protein abundances in drought-stressed M82. **(D)** Venn diagram showing the numbers of up- and down-regulated genes and protein upon drought stress.

The proteomic and mRNA-seq data were combined to reveal the DEGs expressed at the proteomic and mRNA levels. Profiling the mRNA expression provides a global picture of the transcriptional activities in a given system under stress conditions such as drought stress, whereas targeted proteomics identifies the expression abundance of drought stress-related proteins. The correlation coefficient between the transcript abundance and their corresponding proteins under drought stress was 0.088 for ContM82 and 0.093 for DM82. This suggested that the detected genes and proteins were poorly correlated (**Figures 6A, B**), while the DEGs were well correlated with DAPs with R^2 = ^0.743 (**Figure 6C**). Both the transcriptomic and proteomic approaches are important because each provides a unique perspective and opportunities for analyzing the complex drought tolerance mechanisms of tomato.

Furthermore, 169 genes were differentially expressed at transcription and translation levels, among which 112 were up-regulated while 54 were down-regulated (**Figure 6D**). Moreover, three of these genes had varying transcript and protein expression patterns. One of the three genes was up-regulated at the transcript level (Solyc06g006080) but down-regulated at the protein level. The other two genes (Solyc12g099650 and Solyc10g005960) were down-regulated in transcription but up-regulated during translation (**Figure 6D**).

**Figure 7:**
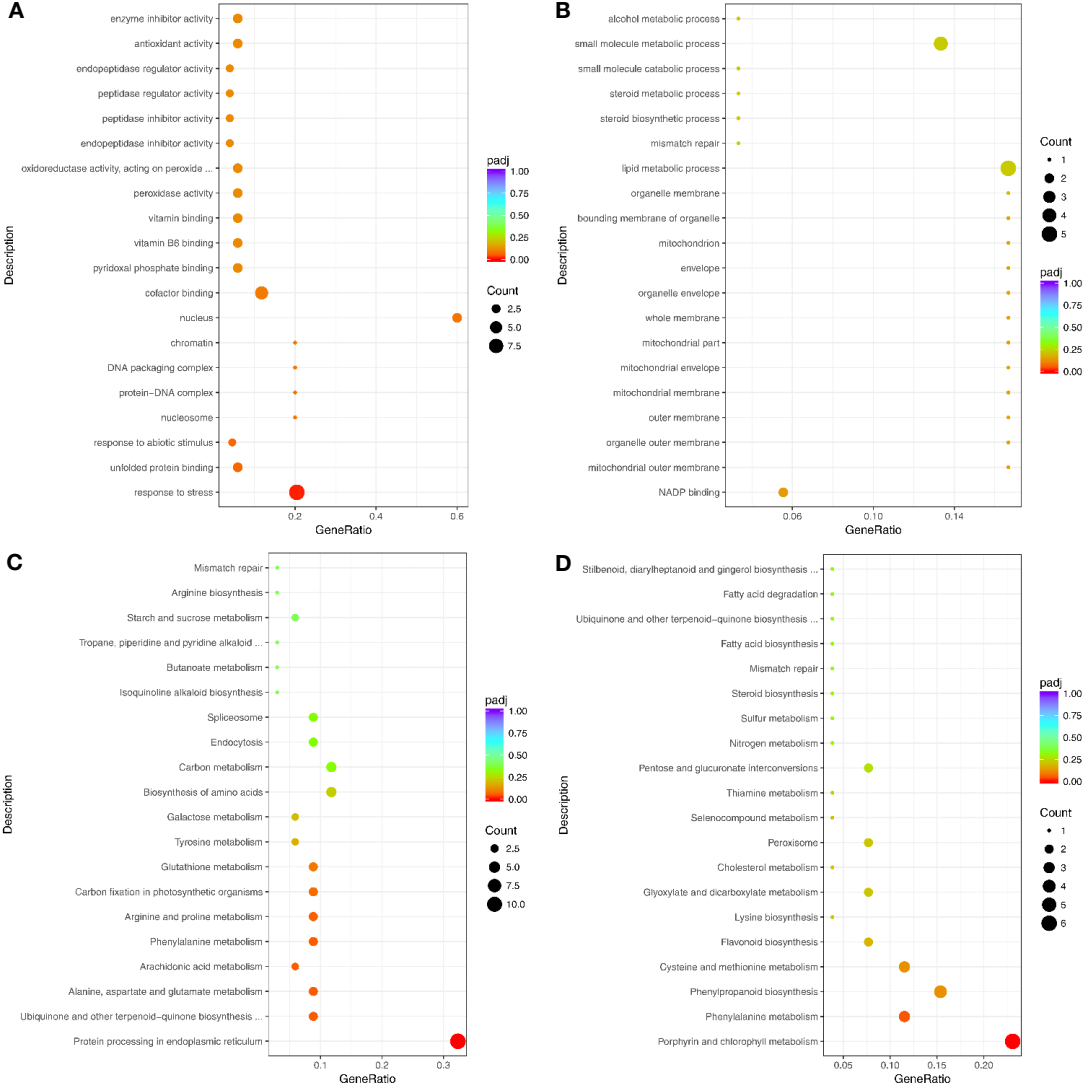
Top 20 GO terms and pathways enriched by the significant DEGs and DAPs under drought stress. **(A)** Up- and **(B)** Down-regulated genes for GO enrichment analysis. **(C)** Up- and **(D)** Down-regulated genes for pathways analysis. Counts indicate the DEGs; The color scale indicates the q value.

The 112 genes up-regulated at both the transcription and translation levels were enriched in 188 GO terms, including 97 biological process terms, 78 molecular function terms, and 13 cellular component terms (**Figure 7A**; **Supplementary Table S16**). The biological process GO terms were mainly related to stress and included response to stress (GO:0006950), response to abiotic stimulus (GO:0055114), and response to oxidative stress (GO:0006979) (**Figure 7A**; **Supplementary Table S16**). Moreover, the molecular function terms were mainly related to oxidoreductase activity, such as peroxidase activity (GO:0004601), oxidoreductase activity (GO:0016491), acting on peroxide as acceptor (GO:0016684), and antioxidant activity (GO:0016209). The 54 genes down-regulated at both transcription and translation levels under drought stress were enriched in 117 GO terms (**Figure 7B**; **Supplementary Table S17**), and only one GO term was significantly enriched in response to stress (GO:0006950).

The 112 up-regulated genes were enriched in 36 KEGG pathways (**Figure 7C**; **Supplementary Table S18**), many of which were related to stress response, including arginine and proline metabolism, glutathione metabolism, and cysteine and methionine metabolism. Interestingly, only one KEGG pathway (involved in protein processing in the endoplasmic reticulum) was significantly enriched. This pathway contained heat shock protein-related genes, including eight Hsp20s (heat shock protein 20) and three Hsp70s. However, the 54 genes that were down-regulated at transcription and translation levels were enriched in 27 pathways, including two significantly enriched pathways (**Figure 7D**; **Supplementary Table S19**). The enriched pathways were mainly involved in growth and development and included metabolic pathways, porphyrin and chlorophyll metabolism, and biosynthesis of secondary metabolites.

A network analysis of the DEGs and DAPs at transcription and translation levels revealed that among the 11 heat shock proteins significantly enriched in pathway enrichment analysis, nine were detected in the same network (**Supplementary Figure S1**).

### Candidate genes that enhance plant resistance to drought stress

**Figure 8:**
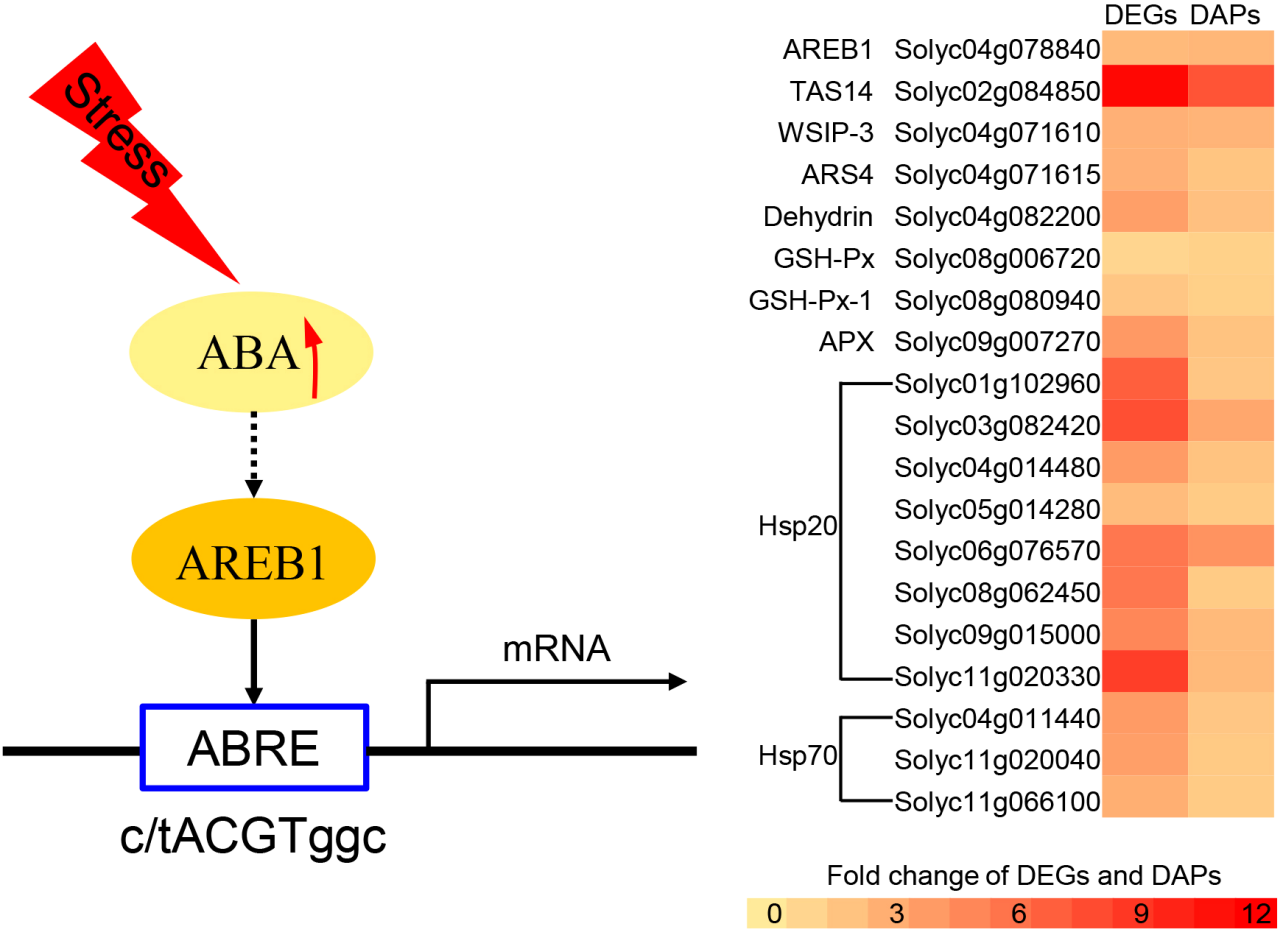
A pathway model of drought stress response in tomato. The fold change of DEGs is Log2 DM82/ContM82. The fold change of DAPs is DM82/ContM82. AREB1: ABA-responsive element binding protein; TAS14: Abscisic acid and environmental stress-inducible protein; WSIP-3: Water-stress inducible protein 3; ARS4: ABA/WDS induced protein; GSH-Px: Glutathione peroxidase; GSH-Px-1: glutathione peroxidase-like encoding 1; APX: Ascorbate peroxidase plant ascorbate peroxidase; Hsp20: Heat shock protein Hsp20; Hsp70: Heat shock protein Hsp70.

**Figure 9:**
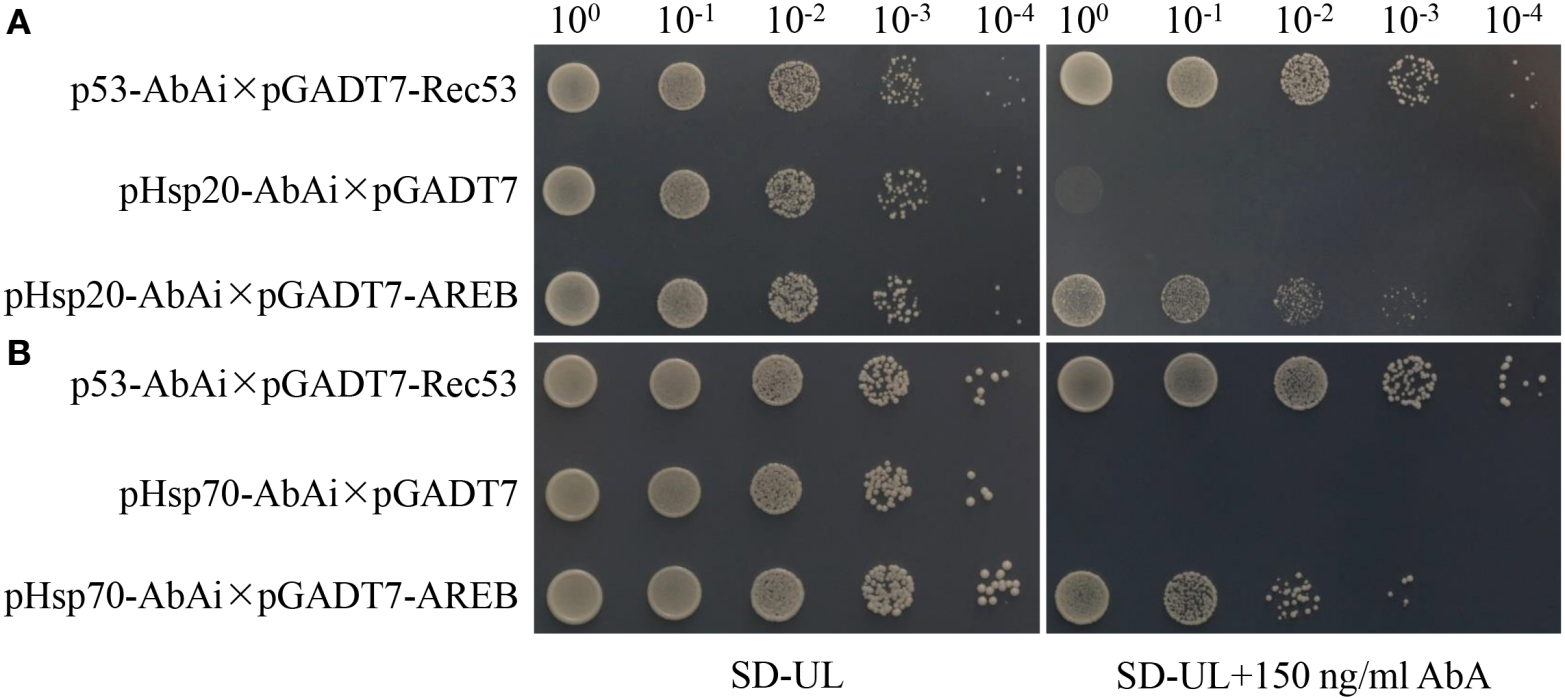
Yeast one-hybrid indicating the interaction between AREB1 proteins and Hsp20 **(A)** and Hsp70 **(B)** promoters. Hsp20: Solyc04g014480; Hsp70: Solyc04g011440.

One of the significantly enriched GO terms (GO:0006950, response to stress) contained nine stress-related genes, seven of which were closely related to drought resistance responses. These included water-stress inducible protein 3 (Solyc04g071610), ASR4 and ABA/WDS induced protein (Solyc04g071615), abscisic acid and environmental stress-inducible protein TAS14 (Solyc02g084850), ascorbate peroxidase (Solyc09g007270), glutathione peroxidase (Solyc08g006720), glutathione peroxidase-like encoding 1 (GSHPx) (Solyc08g080940), and dehydrin (Solyc04g082200). Furthermore, the co-upregulated genes and proteins were significantly enriched in only one KEGG pathway (involved in protein processing in the endoplasmic reticulum), containing 11 heat shock proteins. The promoters of all the 11 genes contained ABRE elements (**Supplementary Figure S2**), among which AREB1 was significantly up-regulated under drought stress (**Figure 8**). Yeast one-hybrid assays revealed that the AREB1 could directly bind the promoter of *Hsp20* (Solyc04g014480) and *Hsp70* (Solyc04g011440) (**Figure 9**). These two genes were significantly enriched in stress-related pathways, such as response to stress and that involved in protein processing in the endoplasmic reticulum. Thus, we speculated that these genes might be closely related to tomato drought resistance.

## Discussion

Drought is among the most important abiotic stresses limiting crop yield and quality worldwide (Farooq et al., 2009; Gupta et al., 2020). With the global climate anomalies, drought has become a key factor restricting the development of agricultural production, necessitating urgent improvement of plant drought resistance. Plant drought tolerance involves a complex regulatory network of multi-layer and multi-gene interactions (Kuromori et al., 2022). Transcriptomic and proteomic approaches are promising in deciphering plant stress tolerance mechanisms and have been successfully applied in studying many plant species (Zhuang et al., 2014; Parihar et al., 2019). However, there are no reports on the use of these approaches in tomato. Different plants have different drought tolerance mechanisms due to the complexity of plant drought responses. The commercial production of tomato, one of the most important vegetable crops worldwide (Sato et al., 2012), is greatly limited by drought stress (Reichardt et al., 2020). Therefore, this study employed high throughput RNA sequencing and protein analysis to identify the molecular mechanism controlling drought tolerance in tomato.

Drought can severely reduce plant growth and development. RWC is an important factor in plant water relations, and its reduction is among the earliest effects of drought stress on plants, which ultimately affects the growth and development of plants (Mishra and Singh, 2010; Pervez et al., 2009). An observed decrease in RWC under drought stress impacted leaf photosynthetic machinery, as evident from reported changes in photochemistry and heat dissipation measured by chlorophyll fluorescence parameters (**Figure 1B**). Drought stress increases the accumulation of ROS, such as H_2_O_2_, O_2_^-^ (superoxide anion), and ·OH (hydroxyl radicals) in plants. The accumulation of ROS can lead to membrane lipid peroxidation, thereby affecting the normal functions of plant cells. We found that the H_2_O_2_ and MDA contents increased significantly in tomato after drought stress, indicating that ROS accumulation caused lipid peroxidation, thus affecting the normal growth and development of tomato. Increased osmotic regulation ability can improve plant drought tolerance (Yamada et al., 2005; Dien et al., 2019). Our study found that increased contents of osmoregulation substances such as proline and soluble sugars could improve tomato drought tolerance. Since the plant hormone ABA plays an important role in drought stress, our results showed that the increased ABA content activated the ABA signaling and improved the drought resistance of tomato. These findings suggest that tomato may have developed a specific drought tolerance mechanism during their long-term evolution.

Sequencing technologies have rapidly developed in recent years, enabling a better understanding of the biological processes of various organisms (Netla et al., 2023). Omics have been widely used to mine stress response genes in plants (Zhuang et al., 2014). RNA-seq can provide qualitative (RNA sequence) and quantitative (RNA abundance) analysis of the targeted mRNA transcripts or complete transcriptome at the tissue level (Stark et al., 2019; Kuksin et al., 2021). Moreover, single-cell RNA sequencing (scRNA-seq) is a relatively new technique that can measure the gene expression of each individual cell in the sample and determine the gene expression pattern of different cell subgroups (Tang et al., 2009; Lähnemann et al., 2020). scRNA-seq has been widely applied in various fields to reveal cell-cell interactions, plant pathogenic interactions, cell-specific gene expression, and regulatory pathways (Kolodziejczyk et al., 2015; Netla et al., 2023). However, this technique has many limitations in analyzing the transcriptomic responses in specific cell types (Netla et al., 2023). For example, scRNA-seq cannot accurately distinguish between the closely related cells at different stages of growth and differentiation (Grün et al., 2015). Improving the separation, amplification, and bioinformatics analysis methods could further improve the reliability of the scRNA-seq data. This study utilized deep sequencing to compare mRNA and protein expression in tomato under normal and drought stress conditions.

This study found that 1527 genes were up-regulated and 2238 were down-regulated in tomato under drought stress, indicating that changes in gene expression could be one of the mechanisms that tomato evolved in response to drought stress. The GO and pathway analyses revealed that most of the significantly down-regulated genes were related to plant growth activities, including nitrogen metabolism (sly00910), photosynthesis (sly00195), response to auxin (GO: 0009733), and enzyme inhibitor activity (GO: 0004857). These results were also observed in previous studies on *Arabidopsis*, which revealed that various mechanisms, including regulation of photosynthesis and stress and defense responses, were enriched in nitrogen metabolism, response to auxin, etc. (Meng et al., 2019). Nitrogen metabolism and photosynthesis are crucial for plant growth (Harris, 1978; Evans, 2013; Iqbal et al., 2020), and auxin is a plant hormone that profoundly affects many aspects of plant development, such as cell division, elongation and differentiation, etc. The GO term, response to auxin, participates in the auxin regulatory pathway. In the present study, the auxin-related terms were significantly enriched in the GO and pathway analysis of the down-regulated DEGs, which might have negatively affected tomato growth under drought stress. Notably, most of the significantly up-regulated genes were involved in response to stress (GO: 0006950), response to abiotic stimulus (GO: 0009628), peroxisome (sly04146), and arginine and proline metabolism (sly00330), which are important for plant drought adaptation (Liu et al., 2017). The gene regulation of these GO terms was involved in drought response, which improved tomato drought tolerance. Thus, these findings highlighted the regulatory mechanisms of tomato drought tolerance.

TMT-based approaches are usually used to analyze the proteins or peptides isolated via the digestion method due to their ability to identify proteins with complex tertiary structures (Zhang and Elias, 2017; Navarrete-Perea et al., 2018). Therefore, such methods are suitable for analyzing the antioxidant enzymes or proteins with complex seasonal structures related to plant drought stress. Alqurashi et al. (2018) utilized TMT quantitative proteomics to profile protein expression in *A. thaliana* under drought stress. The study analyzed 310 of the DEPs and reported that endocytic processes are implicated in early drought stress signaling. Li et al. (2021) also used TMT proteomics to analyze the responses of maize seedlings to mild and severe drought stresses. The results showed that maize could adapt to mild drought by activating the antioxidant system and photorespiration, but severe drought stress damaged the photosynthetic apparatus. In the present study, drought stress significantly up-regulated 165 proteins and down-regulated 129 proteins, suggesting the lack of a one-to-one correspondence between DEGs and DAPs. Earlier studies also reported similar results between transcriptomic and proteomic data (Haider and Pal, 2013; Luo et al., 2018). Tamburino et al. (2017) used 2D-DIGE-MS/MS to investigate the drought-responsive genes in tomato chloroplast and showed that severe drought stress activated a specific retrograde signaling pathway in tomato chloroplast. Chloroplast is an important photosynthesis organelle in green plants (Pan et al., 2021). In this study, nearly half of the down-regulated proteins were located in the chloroplast, indicating that drought stress reduced the photosynthetic activities of tomato, thus affecting their growth and development under drought stress. Plants have self-protective mechanisms which improve their tolerance to drought stress (Zhu, 2016). In this study, the COG/KOG functional classification, GO enrichment and KEGG pathway analyses identified many DAPs involved in the stress-related pathways (**Figures 4A**, **5A, D**), indicating that these DAPs are important in tomato drought tolerance.

Integrating different omics methods is a promising way to understand the multi-level regulation processes in a perturbed system (Lan et al., 2012). This approach has been widely used to study stress responses of many plants, including *Arabidopsis*, maize, soybean, and cotton (Yamaguchi and Sharp, 2010; Lan et al., 2012; Peng et al., 2018). Such integrative approaches would improve the analysis of multiple levels of gene expression to understand the drought response mechanisms since no single approach can fully unravel the complexities of the drought tolerance mechanism of plants. The integrative approach used in this study involved several steps. First, the post-transcriptional processes were evaluated by elucidating how tightly the transcribed mRNA is linked to protein abundance. Secondly, novel aspects of acclimatory processes induced by drought stress were evaluated via a global parallel analysis of protein and mRNA abundance changes. Finally, candidate genes related to drought resistance were identified. Here, the proteomic and transcriptomic data from tomato leaves showed a weak correlation between gene expression and protein abundance. This observation is similar to previous reports on animals and other plants, in which most integrative transcriptomic and proteomic studies showed low correlations (Haider and Pal, 2013; Luo et al., 2018). For example, Luo et al. (2018) revealed a negative correlation (Pearson = 0.09 and 0.40) between protein abundance and gene expression level. Hegde et al. (2003) mentioned “the glass half empty” theory, which indicated that transcriptomics and proteomics were not equivalent. The poor correlation between transcript and protein levels might be due to the impact of translational efficiency factors, such as physical properties of the transcript, the whole structure of the mRNA, codon bias, ribosome density, half-life of eukaryotic mRNA, and variability (normalized standard deviation) of mRNA expression level. Another possibility might be the post-translational (down) regulation of the protein activity to avoid a *de novo* cycle of synthesis after the stress is relieved.

However, this study showed a high correlation between DEGs and DAPs. Hegde et al. (2003) described a “glass half full” theory, which indicated that transcriptomics and proteomics were complementary, each of which provides a unique perspective and synergy for discovering and interpreting biological processes.

Many genes, including TAS14, WSIP-3, ARS4, GSH-Px, APX, Hsp20, and Hsp70 that were differentially expressed at both transcript and protein levels were enriched in the stress-related pathways, which are important in plant drought tolerance (Cho and Choi, 2009; Li et al., 2011; Muñoz-Mayor et al., 2012; Augustine, 2016; Muthusamy et al., 2017; Cunha et al., 2019). Meng et al. (2019) previously conducted a proteomic analysis of *Arabidopsis* under drought stress and found that HSP played an important role in its drought tolerance. Moreover, the genes that were enriched in the stress-related pathways contained ABRE elements in their promoters (**Supplementary Figure S2**), and drought stress significantly increased the ABA content and up-regulated AREB1 at the transcript and protein level. ABA is the main hormone regulating the water balance and osmotic stress responses in plants (Zhu, 2016). Under stress conditions, plants synthesize ABA in various organs to regulate stress responses through various mechanisms, including transcriptional regulation of defense-related genes conferring resistance to drought stress and the ABA metabolism and transport (Fujita et al., 2005; Furihata et al., 2006; Yoshida et al., 2010). A conserved cis-element AREB1 is a key positive regulator of the promoters of such ABA-regulated genes during ABA signaling in vegetative tissues under drought stress (Yoshida et al., 2010). The Hsp family is transcriptionally regulated mostly by heat-shock factors (HSF) (Augustine, 2016). Many studies showed that drought stress up-regulates Hsp20s and Hsp70s (Yu et al., 2016; Muthusamy et al., 2017; Selvi et al., 2020). Zhang et al. (2019) showed that ABA stimulated the accumulation of Hsp. Similarly, our results indicated that ABA regulated Hsp to promote tomato drought tolerance. In addition, the yeast one-hybrid assay demonstrated that AREB1 could directly bind the promoter of *Hsp 20* (*Solyc04g014480*) and *Hsp 70* (Solyc04g011440). These results indicate that drought stress and endogenous ABA activate *AREB1* expression, promoting the expression of heat shock proteins (TAS14 and GSH-Px-1) and ultimately improving the drought resistance of tomato.

## Conclusions

This study shows that drought stress seriously affected the growth and development of tomato by reducing the plant water content, photosynthesis, and redox status. However, the contents of ABA and osmotic regulatory substances such as proline and soluble sugar significantly increased under stress, indicating the specific drought tolerance mechanisms of tomato. Transcriptomic and proteomic analyses showed that 3765 genes and 294 proteins were significantly changed after drought stress. Moreover, the GO and pathway analysis indicated that most of these genes may be involved in stress-related GO terms, such as response to stress, abiotic stimulus, and oxidative stress, suggesting their possible role in tomato drought tolerance. The integrated transcriptomic and proteomic analysis showed that transcriptome and proteome were correlated, highlighting the importance of post-translational events in plant adaptation to drought stress. ABA plays an important role in plant drought tolerance. Our results suggested that drought stress increased the ABA content, which was essential for increasing the expression of AREB1 to regulate heat shock proteins, which confer plant adaptive responses to drought. Thus, these results highlight the drought tolerance signaling network of tomato and provide the knowledge that can be useful in breeding programs of crops.

## Future directions

Drought is one of the most important factors that causes complex and multivariate effects on plant physical and biological characteristics. Moreover, the drought response mechanisms of plants involve complex processes influenced by environmental and genetic backgrounds. Over the next century, global warming will likely increase the severity and frequency of drought events, further limiting the yield and quality of crops. The main challenge in understanding the drought tolerance mechanism of plants is identifying the drought tolerance mechanisms of different species from different environments. Thus, future studies should combine the latest genomics analysis approaches, including quantitative genetics, genomics, and biomathematics, with an ecophysiological basis to identify and verify the functions of key genes involved in drought responses. Moreover, the complex drought tolerance mechanisms of plants should be analyzed to better understand the interaction between crop genotypes and growth environments and provide information for crop improvement.

## Data availability statement

The datasets presented in this study can be found in online repositories. The names of the repository/repositories and accession number(s) can be found below: Name: NCBI. Accession number: PRJNA1003008. https://www.ncbi.nlm.nih.gov/bioproject/PRJNA1003008..

## Author contributions

ML: Conceptualization, Writing – review & editing, Data curation, Formal Analysis, Investigation, Writing – original draft. GZ: Conceptualization, Data curation, Formal Analysis, Investigation, Writing – review & editing. XH: Investigation, Writing – review & editing. TP: Investigation, Writing – review & editing. WC: Investigation, Writing – review & editing. MQ: Investigation, Writing – review & editing. BO: Writing – review & editing, Conceptualization. MY: Conceptualization, Writing – review & editing. SS: Conceptualization, Writing – review & editing.

## Supplementary material

Supplementary Figure 1 The protein-protein interaction network of differentially expressed proteins.

Supplementary Figure 2 The promoters of candidate stress-resistant genes (2 Kb 5′-upstream region of the respective genes), showing the location of ABRE elements.
