# Supplementary figures and images for: Candidate regulators of drought stress in tomato revealed by comparative transcriptomic and proteomic analyses
Source: Front Plant Sci. 2023 Oct 23;14:1282718. doi: 10.3389/fpls.2023.1282718 (PMC10627169; doi:10.3389/fpls.2023.1282718)

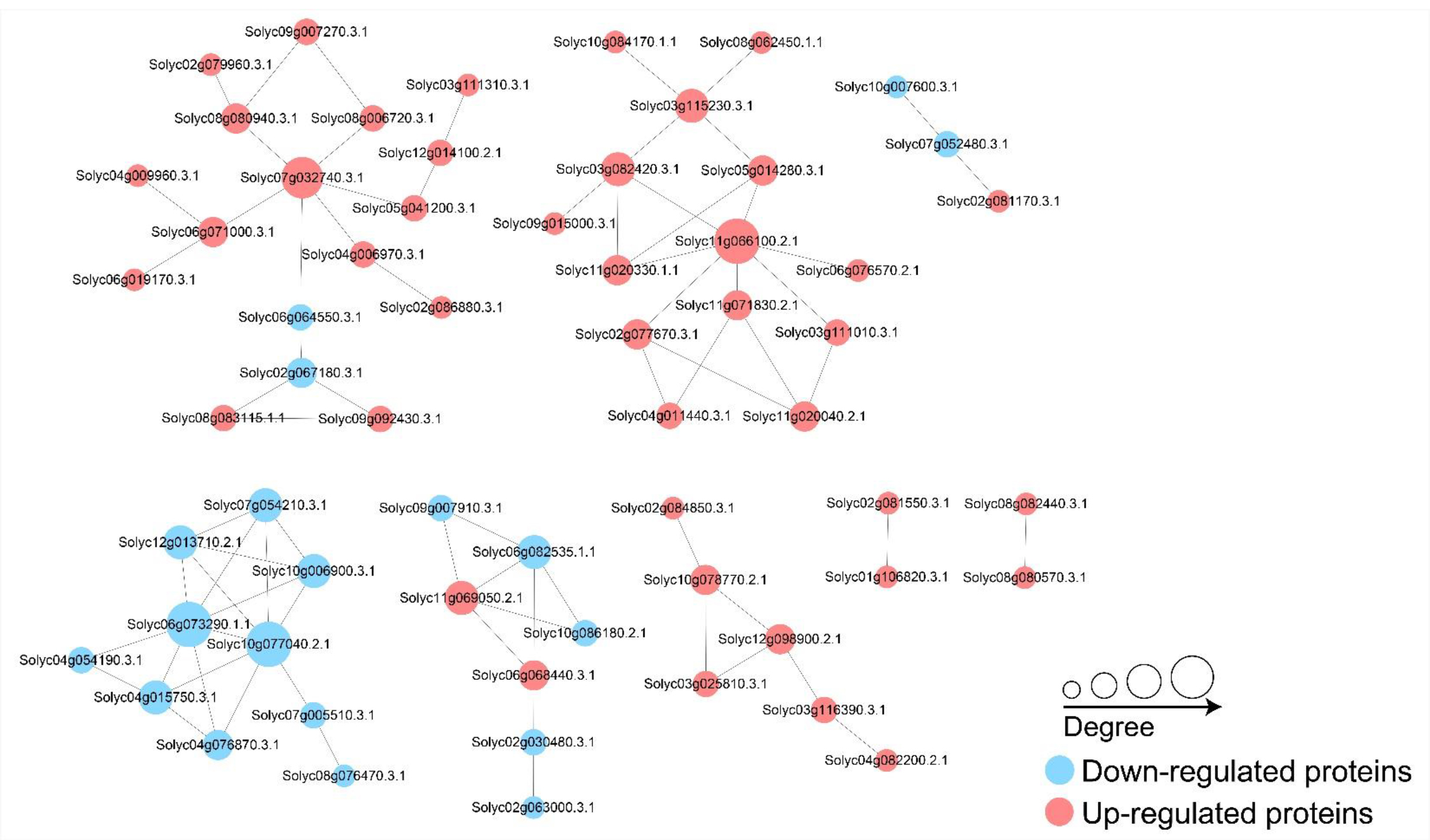

Supplement: Supplementary Figure 1 — The protein-protein interaction network of differentially expressed proteins. [file DataSheet_1.zip › Supplementary Material/Supplementary Figure S1.jpg]

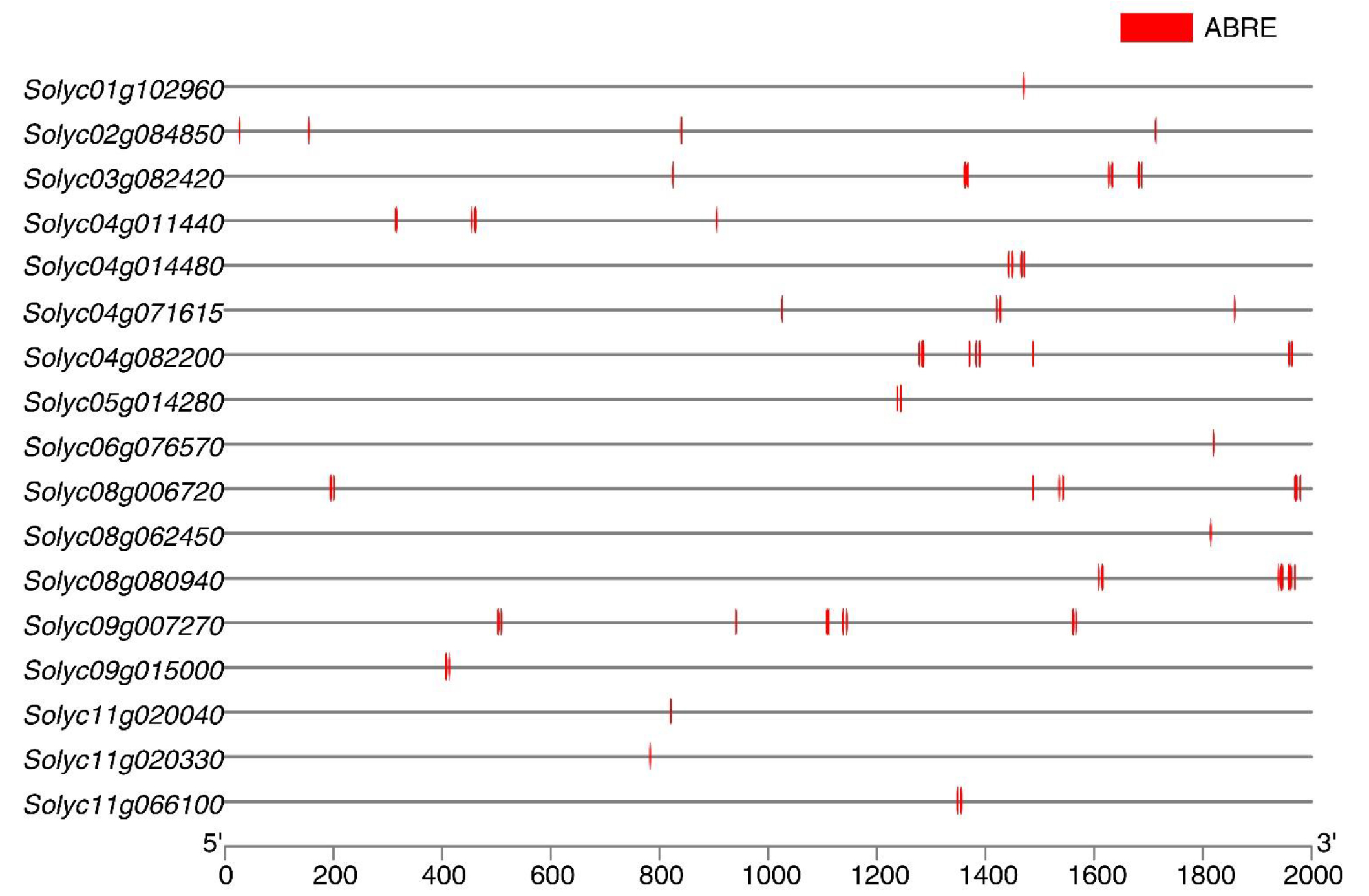

Supplement: Supplementary Figure 1 — The protein-protein interaction network of differentially expressed proteins. [file DataSheet_1.zip › Supplementary Material/Supplementary Figure S2.jpg]
